# Impact of Supplemental Oxygen on Cardiovascular Physiology

**DOI:** 10.3390/cells15100871

**Published:** 2026-05-10

**Authors:** Drithi Chidanand, Rohan Cheruku, Nidhi Sree Perla, Adhira Darapaneni, Siva Kumar Panguluri

**Affiliations:** 1 Department of Pharmaceutical Sciences, Taneja College of Pharmacy, University of South Florida, 12901 Bruce B. Downs Blvd., Tampa, FL 33612, USA; drithichidanand@usf.edu (D.C.); rcheruku@usf.edu (R.C.); nidhisree@usf.edu (N.S.P.); adarapaneni@usf.edu (A.D.); 2 Department of Cell Biology, Microbiology and Molecular Biology, College of Arts and Sciences, University of South Florida, 12901 Bruce B. Downs Blvd., Tampa, FL 33612, USA

**Keywords:** supplemental oxygen, hyperoxia, lung physiology, cardiac remodeling, heart-lung interactions, oxidative stress, mechanical ventilation, reactive oxygen species

## Abstract

Supplemental oxygen is a cornerstone intervention in modern clinical practice, widely used to correct hypoxemia in emergency, perioperative, and critical care settings. While oxygen therapy is lifesaving, accumulating evidence indicates that excessive oxygen exposure can induce significant pathophysiological disturbances, particularly within the cardiovascular and pulmonary systems. Hyperoxia (PaO_2_ > 100 mm Hg) promotes the generation of reactive oxygen species (ROS), leading to oxidative stress, mitochondrial dysfunction, and the activation of pro-fibrotic pathways. When combined with mechanical ventilation, these effects are further amplified through alterations in intrathoracic pressure, reduced venous return, and increased pulmonary vascular resistance, collectively imposing hemodynamic stress on the myocardium. These mechanical and biochemical perturbations converge to drive structural, functional, and electrical remodeling of the heart, including conduction abnormalities and arrhythmogenesis. Emerging clinical insights, particularly from critically ill and COVID-19 populations, underscore the importance of titrated oxygen strategies that balance adequate tissue oxygenation with minimization of hyperoxic injury. This review synthesizes current evidence on hyperoxia-induced oxidative stress, heart-lung interactions, and mechanisms underlying myocardial remodeling to provide a comprehensive framework for optimizing oxygen therapy.

## 1. Introduction

Supplemental oxygen therapy remains one of the most frequently administered interventions in modern medicine and is considered an essential treatment for patients experiencing hypoxemia, respiratory failure, or critical illness [1,2]. By increasing arterial oxygen content, it preserves tissue oxygen delivery and prevents organ dysfunction. Consequently, approximately half of patients admitted to intensive care units receive oxygen therapy at some point during their clinical course [2]. However, oxygen is not biologically inert. Increasing evidence demonstrates that excessive oxygen exposure can disrupt cellular homeostasis and induce systemic toxicity [1,3,4,5,6,7,8,9,10,11,12,13,14,15,16,17,18,19,20,21,22].

A central mechanism underlying oxygen toxicity is hyperoxia, broadly defined as a supraphysiological elevation in arterial oxygen tension, with a PaO_2_ exceeding 100 mmHg conventionally used as the diagnostic threshold [23]. However, its clinical significance is closely tied to severity. Mild hyperoxia (PaO_2_ 100–200 mmHg) may occur transiently during routine oxygen supplementation and is generally tolerated in otherwise healthy individuals. Moderate hyperoxia (PaO_2_ 200–300 mmHg) is associated with progressive oxidative stress, early endothelial dysfunction, and measurable hemodynamic changes. Severe hyperoxia (PaO_2_ > 300 mmHg), most commonly resulting from high fractions of inspired oxygen (FiO_2_) during mechanical ventilation, carries the greatest risk of pulmonary and cardiovascular toxicity and is the range most relevant to the pathophysiological mechanisms discussed in this review. Hyperoxia occurs when oxygen levels in blood and tissues exceed physiological requirements, typically as a result of high fractions of inspired oxygen (FiO_2_) during supplemental oxygen therapy or mechanical ventilation [9]. Under hyperoxic conditions, molecular oxygen undergoes partial reduction, generating reactive oxygen species (ROS) such as superoxide, hydrogen peroxide, and hydroxyl radicals [24]. Excess ROS overwhelms antioxidant defenses, causing oxidative damage and contributing to pulmonary, vascular, and myocardial injury review.

In critically ill patients, the interaction between oxygen therapy and cardiopulmonary physiology is particularly complex. Mechanical ventilation and elevated oxygen concentrations can alter intrathoracic pressure, pulmonary vascular resistance, and ventricular loading conditions, thereby affecting cardiac output and myocardial stress [25]. In addition to hemodynamic changes, hyperoxia promotes inflammatory signaling and mitochondrial dysfunction that may contribute to myocardial remodeling and electrical instability [5]. These processes highlight the interconnected nature of the heart and lungs, where pulmonary injury can propagate systemic effects through inflammatory mediators and oxidative stress pathways.

Recent clinical and experimental studies have therefore shifted attention toward a more conservative and titrated approach to oxygen administration, aiming to maintain adequate oxygenation while minimizing hyperoxic injury [12]. Understanding the physiological consequences of supplemental oxygen on cardiovascular function is essential for optimizing oxygen therapy strategies in critically ill patients.

This review explores the impact of supplemental oxygen and hyperoxia on cardiovascular physiology, with particular emphasis on heart–lung interactions during mechanical ventilation, mechanisms of myocardial structural remodeling, functional hemodynamic alterations, and electrophysiological disturbances associated with excessive oxygen exposure.

## 2. Methods

This article is a narrative review of the current literature regarding the effects of supplemental oxygen of cardiovascular and cardiopulmonary physiology. A comprehensive search was performed via the PubMed database using combinations of the search terms “hyperoxia”, “supplemental oxygen”, and “mechanical ventilation” alongside “lung physiology”, “palliative care”, and “cardiovascular physiology”. Studies were selected based on their direct relevance to cardiopulmonary outcomes, with priority given to randomized controlled trials and high-impact observational studies. To ensure scientific rigor, technical reports and non-peer-reviewed commentaries were excluded from this analysis.

## 3. Supplemental Oxygen

Supplemental oxygen is a cornerstone of emergency and intensive care medicine, primarily utilized to reverse hypoxemia in respiratory failure. While nearly half of all Intensive Care Unit (ICU) patients receive oxygen therapy, the clinical challenge lies in balancing therapeutic benefit against the risks of oxygen toxicity [1,2]. Supplemental oxygen provides a higher concentration than room air to improve gas exchange, with the current guidelines emphasizing a “titrated approach” where delivery methods are chosen based on bedside respiratory assessments such as chest movement and breath sounds [2,26].

### Clinical Delivery and Hemodynamic Impact

Supplemental oxygen delivery follows a stepwise manner starting from a noninvasive (NIV) delivery pattern leading to more invasive techniques depending on the severity of oxygenation needed. Noninvasive oxygen delivery includes nasal cannulas providing a fraction of inspired oxygen (FiO_2_) of 24–40% at flow rates of 1–6 L/min and simple face masks providing an FiO_2_ of 40–60% at flow rates of 5–10 L/min [26]. As flow rate and device support increases, FiO_2_ can be titrated from just above ambient levels to nearly 100% with systems such as non-rebreather masks or high-flow oxygen devices [27]. NIV is used as first-line therapy in emergency departments and out-of-hospital settings for acute chronic obstructive pulmonary disease (COPD)/asthma exacerbations with hypercapnic respiratory failure, pulmonary edema, and hypoxemic failure in immunocompromised patients including post-transplant and cancer, avoiding intubation when possible [28].

Recent clinical guidelines suggest the use of high-flow nasal cannula (HFNC) over conventional oxygen therapy in acute hypoxemic respiratory failure, as it provides physiological benefits like anatomical dead space reduction and heated humidification while improving patient comfort and tolerance. Furthermore, HFNC is recommended during breaks from non-invasive ventilation (NIV) to maintain oxygenation, though clinicians must ensure its use does not delay necessary intubation in deteriorating patients [29]. When oxygenation demands exceed the capabilities of high-flow systems, NIV is considered advantageous because it lowers inspiratory muscle workload, enhances alveolar recruitment, and maintains airway patency while avoiding the complications of mechanical ventilation (MV) [30].

Invasive ventilation on the other hand involves endotracheal intubation or tracheostomy with MV delivering FiO_2_ up to 1.0 (100%), typically combined with positive end-expiratory pressure to support gas exchange in critically ill patients [26]. During MV, such high inspired oxygen fractions can lead to arterial hyperoxia and tissue hyperoxia. Experimental murine models show that supplementing MV with 100% oxygen increases diaphragm vascular resistance and further reduces diaphragmatic blood flow and oxygen delivery compared with normoxic ventilation, providing a mechanistic basis for hyperoxia-related diaphragm injury [8].

Moore et al. demonstrated that supplemental oxygen (50% O_2_) significantly enhanced exercise capacity and reduced exertional breathlessness in patients with chronic congestive heart failure. While these improvements suggested that supplemental oxygen concentrations can acutely alleviate ventilatory and hemodynamic constraints, it is not without cardiovascular cost [31]. Supplemental oxygen is often administered in patients with symptomatic heart failure; however, high concentrations have been shown to acutely reduce cardiac output and stroke volume while increasing pulmonary capillary wedge pressure. These findings suggest that oxygen therapy can impose significant hemodynamic strain, particularly in patients with precariously low baseline cardiac performance [32]. Conversely, inhalation of oxygen at concentrations exceeding 21% induces a vagus-mediated reduction in heart rate, resulting in a rate-dependent decrease in cardiac index [33].

## 4. Hyperoxia

Hyperoxia is a condition in which oxygen levels in tissues and organs become abnormally elevated due to exposure to excessive FiO_2_ or high partial pressure of arterial oxygen (PaO_2_ ≥ 100 mmHg). These highly inspired oxygen fractions produce toxic effects through the excess generation ROS. At high partial pressures, oxygen becomes toxic, with injury most prominently affecting the lung, central nervous system, and eye. The timing and severity of toxicity depends on both dose and duration of exposure [1]. This vulnerability is underscored by the ‘oxygen paradox,’ where the sudden reintroduction of molecular oxygen to oxygen-starved tissues triggers a unique injury response that paradoxically enhances tissue damage beyond the initial period of ischemia [24].

### 4.1. Types of Hyperoxia

Hyperoxia is distinguished between two types. Normobaric hyperoxia refers to exposure to elevated oxygen concentrations at normal atmospheric pressure, typically achieved by administering high FiO_2_ oxygen mixtures through masks or ventilatory support in a standard pressure environment. Hyperbaric hyperoxia is produced during hyperbaric oxygen therapy, where patients breathe near-100% oxygen inside a pressurized chamber set above 1 atmosphere absolute pressure [9].

Normobaric hyperoxia is delivered via nasal cannula or facemask and is widely used as a standard intervention in conditions such as acute ischemic stroke. In stroke, normobaric hyperoxia raises brain tissue oxygen, increasing interstitial partial pressure of arterial oxygen (PaO_2_) in the ischemic penumbra (but not in the infarct core), helping to preserve penumbral tissue without substantially increasing oxidative radical injury in human experimental models [6].

While hyperbaric hyperoxia is used in wound healing measures, it makes oxygen toxicity develop faster than normobaric oxygen due to the increasing amount of oxygen dissolved in blood and tissues, which boosts harmful oxygen radicals and places stress on organs like the lungs and brain. In the lungs, it first causes irritation of the airways with cough and reduced mucus clearance which then leads to lung inflammation, fluid in the lungs, chest pain, and shortness of breath if exposure continues. It also has a clear nerve-related component, with brain and vagus-nerve pathways helping drive symptoms, showing that both direct oxygen damage and nervous-system signals are involved in how the body responds [12].

### 4.2. Systemic Toxicity and Organ Dysfunction

Hyperoxia exerts systemic effects that extend to multiple organs. In the kidney, excessive oxygen administration alters renal blood flow and oxygen homeostasis and has been implicated in the development of acute kidney injury. Interactions of the lung–heart–kidney organs further amplify these effects, so that injury in one organ can influence dysfunction in the other organs under sustained hyperoxia exposure. Beyond these systemic effects, hyperoxia is also a key driver of cardiac remodeling. MV together with prolonged hyperoxia can injure pulmonary epithelial cells [10]. In vivo studies reported that hyperoxia-induced lung injury in mice often led to mortality after approximately 72 to 96 h of exposure evidenced by a 10–15% reduction in total body weight [34]. Hyperoxia was also known to cause elevated QTc and JT intervals, decrease in cardiac output and ejection fraction, bradycardia and cardiac arrhythmias in previous murine studies from our laboratory [5,13,14,15,16,17,18,19,20,21,22].

Despite these risks, hyperoxia also has beneficial effects like supporting postoperative wound healing by providing the high tissue oxygen tensions required for neutrophil-mediated ROS production, phagocytosis, and superoxide-driven bactericidal activity against wound pathogens [4]. Ultimately, supplemental oxygen therapy must be carefully administered to balance therapeutic necessity against the risk of systemic oxidative injury.

## 5. Impact of Supplemental Oxygen on Lung Physiology

Mechanical ventilation serves as a vital therapy for patients suffering from a multitude of different ailments. The primary goal of this intervention is to maintain adequate gas exchange, specifically by ensuring systemic oxygenation and the clearance of carbon dioxide. Central to this process is the titration of the fraction of inspired oxygen (FiO_2_). For decades, the clinical “safety net” leaned toward liberal oxygen administration to prevent the immediate risks of cellular hypoxia. However, an emerging body of evidence suggests that the lungs are exquisitely sensitive to oxygen concentrations, and that “too much” oxygen may be as detrimental as “too little” [35].

Furthermore, the physical presence of high-concentration oxygen within the alveoli alters the mechanical stability of the lung. The “washout” of inert nitrogen, which normally provides a structural scaffold for the air sacs, can lead to absorption atelectasis, effectively reducing the surface area available for gas exchange and creating a paradoxical need for even higher pressures or oxygen levels [36].

For a healthy lung, the biological standard for oxygen in inhaled air is 21%, which is equivalent to the amount of oxygen present in a regular environment. In ventilated patients, maintaining an FiO_2_ between 21% and 41% is generally considered the “safe zone,” provided it maintains adequate systemic oxygenation (between 92–96% for SaO_2_). At these levels, the risk of oxidative stress is minimal. Antioxidants such as glutathione and superoxide dismutase in the lung tissue can easily neutralize the standard production of reactive oxygen species (ROS). N_2_ makes up the bulk of the gas at these levels, acting as a “stent” that keeps the alveoli open at the end of expiration [36].

Within the 41% to 60% range for FiO_2_, the partial pressure of nitrogen within the alveoli decreases. Oxygen is rapidly sequestered by pulmonary capillary hemoglobin, and if the rate of oxygen uptake exceeds the rate of alveolar ventilation, the alveolar volume decreases, leading to alveolar collapse. The 41–60% range has been shown to blunt the hypoxic pulmonary vasoconstriction (HPV) reflex. HPV is a homeostatic mechanism that diverts blood flow away from poorly ventilated lung units toward well-ventilated ones [37]. By increasing alveolar oxygen tension in poorly ventilated areas without necessarily improving their gas-exchange capacity, moderate FiO_2_ levels can cause vasodilation in these “shunt” areas. This redistribution of blood flow can lead to an increase in the venous admixture and a decrease in the efficiency of gas exchange. Animal research for low-to-moderate hyperoxia, defined as FiO_2_ between 21% and 60%, has shown an increase in lung lavage protein concentrations, suggesting early injury [11]. Studies also found that exposure to FiO_2_ of 50% to 60% for 7 days alone decreased survival time when animals were subsequently exposed to 100% oxygen, suggesting that moderate hyperoxia “primes” the lung for more severe toxicity [11]. In models involving an inflammatory challenge (infectious and noninfectious) low-to-moderate FiO_2_ consistently increased standardized mean differences in lung weights—an indication for pulmonary edema and fluid accumulation [11].

Clinical literature frequently identifies a FiO_2_ of 0.60 as the pivotal threshold where the risk of pulmonary oxygen toxicity begins to outweigh the benefits of supplemental oxygenation. Sustained exposure to 60% FiO_2_ overwhelms the lung’s endogenous antioxidant defenses, leading to an accelerated production of reactive oxygen species (ROS) such as superoxide and hydrogen peroxide. These ROS directly attack the lipids and proteins within the cell membranes of Alveolar Type I and Type II cells, a process termed lipid peroxidation [7]. This cellular damage specifically impairs the production of surfactants, which increases surface tension and reduces lung compliance, making the lungs “stiff” and harder to ventilate. Beyond chemical damage, high concentrations of oxygen exert a mechanical effect known as absorption atelectasis [36]. In a normal environment, nitrogen provides a structural scaffold that keeps the alveoli open because it is not easily absorbed into the blood. When FiO_2_ increases to 60% or higher, oxygen replaces this nitrogen; as the oxygen is rapidly absorbed into the bloodstream, the alveoli lose internal pressure and collapse. This nitrogen “washout” reduces the functional surface area for gas exchange and can lead to hyperoxic acute lung injury (HALI), a condition pathologically similar to acute respiratory distress syndrome (ARDS) characterized by non-cardiogenic edema and, eventually, pulmonary fibrosis [3].

## 6. Supplemental Oxygen Induced Cardiac Pathophysiology

### 6.1. Mechanical Ventilation as a Structural Stressor of the Myocardium

**Figure 1:**
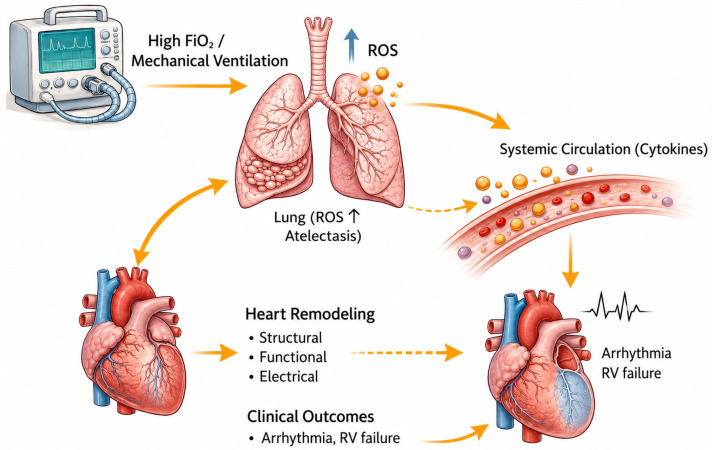
Heart–lung–ROS axis in hyperoxia-induced cardiovascular remodeling: Excessive oxygen administration during mechanical ventilation increases reactive oxygen species (ROS) production within the lungs, contributing to epithelial injury and absorption atelectasis. Pulmonary oxidative stress and inflammation promote the systemic release of cytokines, including TNF-α and IL-6, into the circulation. These circulating inflammatory and oxidative mediators contribute to cardiac remodeling through structural changes (fibrosis and hypertrophy), functional impairment (reduced cardiac output and increased right ventricular afterload), and electrophysiological disturbances associated with arrhythmogenesis. Collectively, these mechanisms contribute to adverse clinical outcomes, including ventricular arrhythmias, right ventricular failure, and multi-organ dysfunction.

Mechanical ventilation (MV) alters the intrathoracic pressure environment and thereby directly modifies cardiac loading conditions [25]. During spontaneous breathing, negative intrathoracic pressure enhances venous return by increasing the pressure gradient between the systemic venous system and the right atrium [37,38,39]. In contrast, positive-pressure ventilation elevates intrathoracic and right atrial pressures, which reduce this gradient and limit venous return [40,41]. These changes are more accurately described in terms of transmural pressure which is defined as the difference between intracavitary and surrounding thoracic pressures [42]. As myocardial wall tension depends on transmural rather than absolute intracavitary pressure, increases in intrathoracic pressure reduce effective cardiac loading even when systemic arterial pressure remains unchanged [43]. Consequently, mechanical ventilation imposes cyclic alterations in myocardial wall stress with each respiratory cycle, modifying strain patterns and activating mechanosensitive signaling pathways involved in structural remodeling [44]. Thus, beyond transient effects on cardiac output, mechanical ventilation fundamentally reshapes the mechanical forces governing myocardial stress and contributes to long-term structural adaptation (Figure 1).

Mechanical ventilation also exposes the myocardium to repetitive mechanical strain, to which cardiomyocytes respond through intrinsic mechanosensitive signaling pathways [45]. Stretch-sensitive ion channels and cytoskeletal elements transduce alterations in wall stress into intracellular biochemical signals [46]. Increases in ventricular wall stress, whether driven by elevated afterload or chamber dilation, activate signaling cascades such as MAP kinase, calcineurin–NFAT, and PI3K–Akt, leading to changes in gene transcription [47]. Molecular data derived primarily from animal models suggest that these transcriptional adaptations promote synthesis of contractile proteins and sarcomeric expansion, increasing cardiomyocyte cross-sectional area [48]. With sustained mechanical loading, these processes shift from adaptive responses to persistent hypertrophic remodeling, encoding mechanical stress as a structural phenotype within the myocardium [49].

This mechanotransduction response is particularly pronounced in the right ventricle due to its sensitivity to afterload [50]. Pulmonary vascular resistance (PVR) is highly dependent on lung volume and alveolar pressure; during mechanical ventilation, elevated positive end-expiratory pressure (PEEP) and alveolar overdistension compress pulmonary capillaries increases resistance within the pulmonary circulation [51,52,53]. As PVR rises, the right ventricle must generate higher pressures to maintain forward flow; leading to increased wall stress in accordance with Laplace’s law [54]. Initially, the right ventricle compensates through enhanced contractility and hypertrophic remodeling to normalize wall tension [55]. However, with sustained afterload, this adaptive response becomes maladaptive, resulting in chamber dilation that further increases in wall stress and progressive mechanical inefficiency [56].

The structural consequences of right ventricular remodeling extend beyond the right ventricle through ventricular interdependence [57]. As right ventricular pressure rises and dilation develops, the septal displacement toward the left ventricle alters ventricular geometry, reducing left ventricular diastolic compliance and redistributing myocardial wall stress [58,59]. Subendocardial regions may be particularly affected, as sustained mechanical strain activates hypertrophic and fibrotic signaling pathways within the left ventricle, even in the absence of primary LV pathology [60]. Consequently, pulmonary vascular stress induced by mechanical ventilation can secondarily drive left ventricular remodeling through mechanical coupling.

Neurohormonal activation further contributes to structural remodeling during mechanical ventilation [52]. Patients requiring ventilatory support often exhibit increased sympathetic activity and activation of the renin–angiotensin–aldosterone system (RAAS) [61]. Sustained catecholamine exposure increases intracellular calcium cycling and metabolic demand within cardiomyocytes, promoting hypertrophy and, with chronic stimulation, apoptotic signaling pathways [62]. Angiotensin II contributes by stimulating hypertrophic gene expression through AT1 receptor activation and by promoting fibroblast-mediated extracellular matrix production, while aldosterone enhances collagen synthesis within the myocardial interstitium [63,64]. The net effect is progressive extracellular matrix expansion and increased myocardial stiffness [65]. Collagen deposition reduces ventricular compliance, impairs diastolic relaxation, elevates filling pressures, and alters myocardial wall stress distribution, reinforcing maladaptive remodeling and predisposing to functional and electrical instability [66,67].

Inflammatory signaling from ventilator-induced lung injury represents an important pathway through which mechanical ventilation affects myocardial structure [68]. Exposure of alveoli to excessive mechanical stretch or high oxygen concentrations leads to the release of pro-inflammatory mediators into the systemic circulation, extending the effects of lung injury beyond the lungs [69]. Circulating cytokines such as tumor necrosis factor-α and interleukin-6 interact with receptors on cardiomyocytes and cardiac fibroblasts, activating intracellular signaling pathways including nuclear factor κB (NF-κB) and related transcriptional regulators [70,71]. These signaling pathways alter gene expression within cardiac tissue, increasing the expression of genes associated with hypertrophy and extracellular matrix production while reducing those involved in normal contractile function [72]. This shift promotes fibroblast activation and collagen deposition within the myocardial interstitium, contributing to expansion of the extracellular matrix [73]. As collagen accumulates, myocardial stiffness increases, disrupting the structural organization of cardiomyocytes and impairing coordinated contraction [74,75]. These changes reduce ventricular compliance, impair diastolic relaxation, and elevate filling pressures, further altering myocardial wall stress distribution, and promoting ongoing structural remodeling [74,76]. In this way, inflammatory signaling originating in the lung contributes to progressive remodeling of the myocardium during mechanical ventilation.

Oxidative stress further intensifies this structural remodeling process. Hyperoxia, which is frequently employed during mechanical ventilation to ensure adequate arterial oxygenation, increases the generation of reactive oxygen species (ROS) in both pulmonary and systemic tissues [77]. Within cardiomyocytes, oxidative stress damages mitochondrial membranes and impairs components of the electron transport chain [78]. As mitochondrial efficiency declines, oxidative phosphorylation becomes less effective, and ATP production decreases [79].

Energy availability is critical for myocardial function. Actin–myosin cross-bridge cycling during contraction requires ATP, as does calcium reuptake into the sarcoplasmic reticulum during relaxation [80]. When ATP production is impaired, relaxation becomes energetically inefficient, and diastolic function deteriorates [81]. Chronic energetic stress also activates compensatory signaling pathways that promote structural remodeling and hypertrophic adaptation [82]. In this way, oxidative injury links metabolic stress to architectural change.

Reactive oxygen species additionally influence extracellular matrix remodeling directly. Oxidative signaling activates pro-fibrotic transcription factors and promotes differentiation of fibroblasts into myofibroblasts, the cells responsible for collagen deposition [83]. Increased myofibroblast activity accelerates interstitial fibrosis and stiffening of the ventricular wall. Beyond the interstitium, oxidative stress affects the coronary microvasculature. ROS reduces nitric oxide bioavailability and impairs endothelial function [84]. Endothelial dysfunction compromises coronary microcirculation, limiting oxygen delivery at the tissue level. Subclinical microvascular ischemia may develop, which further stimulates inflammatory and fibrotic signaling cascades [85]. Creating a reinforcing cycle in which oxidative stress results in fibrosis, and fibrosis impairs perfusion, leading to additional oxidative stress.

The duration of exposure plays an important role in determining whether these responses remain adaptive or progress toward maladaptive remodeling [86]. Short-term mechanical ventilation may produce transient changes in preload and afterload without lasting structural effects. In contrast, sustained pulmonary vascular stress, persistent inflammatory activation, chronic neurohormonal signaling, and ongoing oxidative injury establish a feed-forward pattern of remodeling [87]. As hypertrophy progresses and extracellular matrix content increases, chamber geometry changes, ventricular walls may thicken or dilate, and compliance decreases, leading to impaired diastolic filling [88]. Once interstitial fibrosis is established, reversal becomes limited, as collagen deposition reflects structural reorganization rather than a transient cellular response [89]. Prolonged mechanical ventilation may therefore leave a persistent structural imprint on the myocardium even after respiratory support is discontinued [90].

Overall, mechanical ventilation influences cardiac structure through interacting mechanical, vascular, neurohormonal, inflammatory, and oxidative mechanisms [52]. These processes act across multiple levels, linking altered loading conditions to changes in gene expression, mitochondrial function, and extracellular matrix composition. The result is progressive remodeling characterized by increased stiffness, altered chamber configuration, and reduced mechanical efficiency, providing a substrate for subsequent functional impairment and electrical instability [71,74].

### 6.2. Functional Cardiac Consequences of Mechanical Ventilation

Mechanical ventilation alters cardiac function in clinically significant ways, as cardiac output is the primary determinant of systemic oxygen delivery [40]. Even with adequate oxygenation, reduced cardiac output can result in impaired tissue perfusion and hypoxia [91]. Therefore, the effects of mechanical ventilation must be evaluated in the context of both respiratory and circulatory function [92]. In critically ill patients, modest reductions in preload or increases in right ventricular afterload can substantially decrease systemic perfusion, exacerbate shock, and contribute to organ injury [92]. Functionally, mechanical ventilation imposes cyclical changes in cardiac loading conditions, altering ventricular filling and ejection on a breath-by-breath basis [93]. Ventilator settings can therefore influence cardiac performance depending on the underlying physiological state [93].

Venous return is determined by the pressure gradient between mean systemic filling pressure and right atrial pressure [94]. During positive-pressure ventilation, increases in intrathoracic pressure raise right atrial pressure and reduce this gradient, limiting venous return [41,95]. As preload declines, stroke volume falls through the Frank–Starling relationship, leading to reductions in cardiac output [96]. These effects are particularly pronounced in volume-depleted states, where baseline filling pressures are already low and relatively small increases in intrathoracic pressure produce disproportionately large reductions in venous return [41]. Under these conditions, patients with sepsis, dehydration, bleeding, or aggressive diuresis may develop hypotension or impaired perfusion when PEEP or mean airway pressure is increased [97]. Reduced venous return lowers cardiac output and oxygen delivery, and if oxygen delivery falls below metabolic demand, tissue hypoxia and organ dysfunction may occur even when pulmonary oxygenation appears adequate [98,99]. This can create a mismatch in which respiratory parameters improve while systemic perfusion worsens, emphasizing the need to interpret ventilator changes within a hemodynamic context [99].

In addition to reducing preload, mechanical ventilation can increase right ventricular (RV) afterload through elevations in pulmonary vascular resistance. Pulmonary vascular resistance is closely related to lung volume and alveolar pressure, with alveolar overdistension compressing pulmonary capillaries and poorly ventilated regions contributing through hypoxic vasoconstriction [100,101,102]. As resistance rises, the right ventricle must generate higher pressures to maintain forward flow. Although the RV may initially compensate through increased contractility, sustained afterload can lead to reduced stroke volume and chamber dilation, increasing wall stress, oxygen demand, and mechanical inefficiency [52,103,104]. Because right ventricular output determines left ventricular preload, reductions in forward flow can secondarily impair left ventricular filling and reduce systemic cardiac output [105]. These interactions highlight the importance of right ventricular function during mechanical ventilation, as ventilator settings can influence pulmonary vascular resistance and contribute to circulatory instability in susceptible patients [97,106].

Venous return is determined by the pressure gradient between mean systemic filling pressure and right atrial pressure [94]. During positive-pressure ventilation, increases in intrathoracic pressure raise right atrial pressure and reduce this gradient, limiting venous return [41,95]. As preload declines, stroke volume falls through the Frank–Starling relationship, reducing cardiac output [96]. These effects are particularly pronounced in volume-depleted states, where baseline filling pressures are low and small increases in intrathoracic pressure produce disproportionately large reductions in venous return [41]. As a result, patients with sepsis, dehydration, bleeding, or aggressive diuresis may develop hypotension or impaired perfusion when PEEP or mean airway pressure is increased [97]. Reduced venous return lowers cardiac output and oxygen delivery, and if delivery falls below metabolic demand, tissue hypoxia and organ dysfunction may occur despite preserved pulmonary oxygenation [98,99].

In addition to reducing preload, mechanical ventilation can increase right ventricular (RV) afterload through elevations in pulmonary vascular resistance. Pulmonary vascular resistance is sensitive to lung volume and alveolar pressure, with alveolar overdistension compressing pulmonary capillaries and poorly ventilated regions contributing through hypoxic vasoconstriction [100,101,102]. As resistance rises, the right ventricle must generate higher pressures to maintain forward flow. Although the RV may initially compensate, sustained afterload can reduce stroke volume and lead to dilation, increasing wall stress and reducing mechanical efficiency [52,104]. Because right ventricular output determines left ventricular preload, impaired forward flow can reduce left ventricular filling and systemic cardiac output [105]. These effects highlight the importance of right ventricular function during mechanical ventilation, as ventilator settings can contribute to circulatory instability by increasing afterload and limiting forward flow [97,106]. Prolonged increases in right ventricular wall stress may also contribute to ventricular remodeling and impaired ventricular interdependence, further limiting left ventricular filling and systemic perfusion [107,108,109,110,111]. Elevated pulmonary pressures and right ventricular strain during mechanical ventilation have additionally been associated with adverse hemodynamic effects and poorer clinical outcomes in critically ill patients [112,113,114,115].

Diastolic function is an important determinant of cardiac performance during mechanical ventilation [116]. Increases in intrathoracic pressure and reductions in preload result in lower ventricular filling volumes. In patients with reduced compliance due to hypertrophy, fibrosis, or inflammation, small reductions in filling can lead to disproportionate declines in stroke volume [39]. Alterations in calcium handling may further impair relaxation and increase diastolic filling pressures [117]. Under these conditions, mechanical ventilation can unmask or exacerbate diastolic dysfunction by limiting filling reserve [116]. As a result, cardiac output may become restricted despite preserved systolic function, particularly in populations in whom diastolic dysfunction is common and frequently under-recognized [110,118].

Echocardiography provides insight into cardiac function during mechanical ventilation but must be interpreted in the setting of altered loading conditions [39]. Ejection fraction is load-dependent and may appear preserved or elevated due to reductions in afterload, even when stroke volume is reduced [25]. A normal or hyperdynamic ejection fraction can therefore coexist with impaired forward flow when ventricular filling is limited [13,17,21,119]. Diastolic indices may also shift with changes in intrathoracic pressure and venous return, rather than reflecting intrinsic abnormalities in relaxation alone [120]. Stroke volume, left ventricular outflow tract velocity–time integral (LVOT VTI), and indices of right ventricular function, including tricuspid annular plane systolic excursion (TAPSE) and septal motion, provide a more direct assessment of hemodynamic status [39,110]. Right ventricular dilation, septal flattening, and reduced forward flow can indicate pressure overload and ventricular interdependence. Respiratory variation in flow measurements and evidence of elevated filling pressures further reflect the impact of mechanical ventilation on cardiac performance.

Mechanical ventilation alters cardiac function by changing the pressure environment of the thorax and the resistance against which the right ventricle pumps [25]. These effects influence venous return, right ventricular afterload, ventricular interaction, and ultimately cardiac output [41]. Reductions in cardiac output can impair organ perfusion and oxygen delivery, contributing to worsening shock physiology and multi-organ dysfunction even when oxygenation improves [121]. The cardiovascular effects of mechanical ventilation therefore represent a central component of ventilator management, particularly in patients with ARDS, sepsis, limited preload reserve, or impaired right ventricular function [97].

### 6.3. Electrical Remodeling During Mechanical Ventilation

The electrical activity in the myocardium depends on the coordinated activation and inactivation of ion channels embedded within the cardiomyocyte membrane [122]. The initiation of each heartbeat begins with a rapid depolarization phase, followed by a precisely timed repolarization process that restores membrane stability before the next excitation cycle [122]. The integrity of this sequence depends on both the density of available ion channels and the kinetics governing their gating behavior [123]. Mechanical ventilation does not directly impose electrical stimulation on the heart; however, it alters the physiologic environment in which ion channels function [124]. The combination of systemic inflammation, oxidative stress, mechanical strain, and temperature shifts progressively modifies ion channel expression and conductance [125]. Thus, mechanical ventilation indirectly reshapes the electrophysiologic landscape of the myocardium [124].

The rapid upstroke of the ventricular action potential is mediated by the fast inward sodium current (*I_Na_*), conducted primarily through Nav1.5 channels encoded by SCN5A [126]. The magnitude of this sodium current determines the slope of phase for depolarization and therefore establishes conduction velocity across myocardial tissue [127]. Efficient propagation requires a sufficient density of functional sodium channels at the sarcolemmal membrane and rapid channel activation kinetics [128]. During prolonged mechanical ventilation, systemic inflammatory mediators such as TNF-α and IL-6 become elevated [128,129]. The binding of these cytokines to myocardial receptors activates intracellular signaling pathways that regulate ion channel gene transcription [130]. Sustained inflammatory signaling has been shown to reduce sodium channel expression and impair channel trafficking to the membrane [131]. Due to this reduction in channel availability, peak sodium current declines [132].

The decrease in sodium current slows the rate of membrane depolarization and reduces conduction velocity [133]. The slowing of impulse propagation produces spatial heterogeneity in electrical conduction across the ventricular myocardium [127]. Thus, wavefronts may encounter regions of partially recovered excitability, allowing reentrant circuits to form [134]. Reentry represents one of the principal mechanisms underlying sustained ventricular tachyarrhythmias [135]. Therefore, even moderate sodium channel downregulation during mechanical ventilation can create a conduction substrate vulnerable to arrhythmia [136].

The repolarization phase of the action potential is governed primarily by outward potassium currents, including *I_Kr_*, *I_Ks_*, *I_K1_*, and *I_to_* [137]. The duration of repolarization determines the refractory period and coordinates recovery of excitability across myocardial tissue [138]. Uniform repolarization ensures synchronized ventricular relaxation and prevents premature re-excitation [139]. Inflammatory mediators and reactive oxygen species directly influence potassium channel expression and conductance [140]. The oxidative modification of channel proteins alters their gating properties, while cytokine-driven transcriptional changes may reduce potassium channel subunit synthesis [141]. Thus, outward repolarizing current density declines [142].

**Figure 2:**
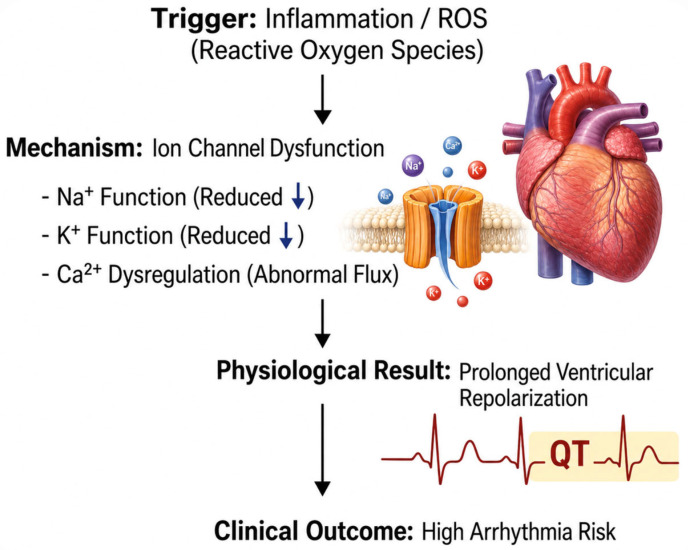
Electrophysiological remodeling under hyperoxic and inflammatory stress: Inflammation and oxidative stress alter cardiomyocyte ion channel expression and function. Downregulation of sodium channels (Nav1.5) slows conduction velocity, while reduced potassium currents prolong repolarization, manifesting as QT interval prolongation. Concurrent calcium handling abnormalities promote delayed and early afterdepolarizations. Together, these changes create a substrate for reentrant circuits and triggered activity, increasing susceptibility to malignant arrhythmias.

The reduction in potassium current prolongs action potential duration and extends the refractory period [143]. On surface electrocardiography in clinical settings, this manifests as QT interval prolongation [144]. However, the more critical effect occurs at the tissue level. Repolarization does not prolong uniformly; instead, regional differences in recovery time develop [145]. This dispersion of refractoriness creates voltage gradients that facilitate abnormal impulse propagation [146]. Due to prolonged repolarization, early afterdepolarization may arise during phase 2 or phase 3 of the action potential [147]. Our laboratory being pioneer in hyperoxia-induced cardiac electrical and structural remodeling reported significant increase in QTc intervals along with brady-arrythmias in both ages and sexes [13,14,15,17,20,21]. In mechanically ventilated patients receiving QT-prolonging medications, this effect may be amplified [148]. Figure 2 illustrates impact of supplemental oxygen/hyperoxia on cardiac electrophysiology and thereby arrhythmias.

The stability of myocardial excitation also depends on tightly regulated intracellular calcium cycling [149]. The entry of calcium during the action potential triggers further calcium release from the sarcoplasmic reticulum, initiating contraction [150]. The reuptake of calcium into the sarcoplasmic reticulum during diastole requires ATP-dependent pumps, primarily SERCA [151]. Oxidative stress impairs SERCA function and increases ryanodine receptor leakiness [152]. Due to impaired reuptake and increased spontaneous release, intracellular calcium homeostasis becomes unstable [153].

The spontaneous release of calcium during diastole activates the sodium–calcium exchanger, generating inward depolarizing current [154]. This inward current produces delayed afterdepolarizations [154]. Delayed afterdepolarizations serve as electrical triggers capable of initiating premature ventricular contractions [155]. The presence of heightened sympathetic activity during critical illness further increases intracellular calcium load [156]. Thus, the probability of triggered depolarizations rises in mechanically ventilated patients [157]. When such triggers occur in myocardium characterized by slowed conduction and repolarization dispersion, sustained arrhythmias may develop [158].

Mechanical stretching introduces an additional mechanism of electrical instability. The increase in right ventricular afterload during mechanical ventilation elevates ventricular wall tension [101]. The stretch of cardiomyocyte membranes activates mechanosensitive ion channels [157]. The activation of these channels generates depolarizing currents that lower the excitation threshold [159]. Thus, mechanical stress can directly provoke electrical activity independent of inflammatory or oxidative influences [159]. This phenomenon, termed mechano-electric feedback, demonstrates the integration between structural load and membrane excitability [160].

Temperature further modulates ion channel kinetics. The opening and closing of sodium and potassium channels involve conformational protein transitions that are temperature-dependent [161]. The elevation of body temperature accelerates channel activation and inactivation rates [162]. Fever, common in critically ill ventilated patients, alters action potential morphology [163]. Due to regional differences in channel expression and metabolic stress, temperature shifts may increase repolarization heterogeneity. Thus, fever does not merely increase heart rate; it modifies the balance between depolarizing and repolarizing currents at the molecular level [145].

Arrhythmogenesis requires both a trigger and a vulnerable substrate [164]. The triggers during mechanical ventilation include delayed afterdepolarizations arising from calcium dysregulation and stretch-induced depolarizations [159]. The substrate consists of slowed conduction from sodium channel dysfunction and repolarization dispersion from potassium channel remodeling [165]. Structural fibrosis, when present, further disrupts electrical continuity [166]. Thus, mechanical ventilation creates an environment in which mechanical strain, inflammatory signaling, oxidative injury, metabolic stress, and temperature modulation converge upon ion channel systems [167]. This convergence produces cumulative electrophysiologic instability rather than a single isolated disturbance [167].

### 6.4. Supplemental Oxygen Exacerbates Cardiac Injury in COVID-19 Patients

While mechanical ventilation has been integral in the treatment of COVID-19 since its beginnings, longitudinal clinical data demonstrates invasive mechanical ventilation exacerbates and accelerates cardiac remodeling. Extensive data from the CARDIO COVID 20-21 registry [168] indicates that COVID-19 patients with myocardial infarction (MI) during their hospitalization were significantly more likely to have received invasive mechanical ventilation (*p* < 0.001). These patients also had greater odds of cardiopulmonary hospitalizations after they recovered from the initial COVID-19 illness, even when adjusting for other factors. In addition, Latin American cohort studies reported high mortality in patients with myocardial injury who received mechanical ventilation. In fact, except for being more than 80 years old, invasive mechanical ventilation was the single biggest risk factor for in-hospital mortality among these cohorts of COVID-19 patients, even among other risk factors such as chronic kidney disease [169]. Another piece of compelling evidence for cardiac injury during COVID-19 infection being exacerbated by mechanical ventilation is the timeline of de novo (new onset) cardiac injury that only occurred after intubation. In one study where echocardiograms were conducted during serial visits of critically ill COVID-19 patients, it was found that 31% of patients with no pre-existing right ventricular injury developed *de novo* RV dysfunction after being intubated and placed on mechanical ventilation [170]. These studies collectively evidence worse cardiac outcomes for COVID-19 patients receiving mechanical ventilation.

One potential mechanism to explain this pattern is continuous high positive end expiratory pressure (PEEP) from invasive mechanical ventilation artificially raising pressure in the thorax, compressing alveolar capillaries, and increasing pulmonary vascular resistance [171]. This, in turn, increases the stress on the thin-walled right ventricle, causing progressive decline. As time passes on the ventilator, COVID-19 patients show structural damage, such as progressive RV dilation and uncoupling initially absent during intubation [172]. The longer the lungs are compromised by high pressure, the greater the effect on the heart.

Ultimately, the combination of mechanical ventilation and COVID-19 infection produces a potent synergy that exacerbates cardiac remodeling and injury. The right ventricle is mechanically overloaded by the pressure from the ventilator, causing mechanical strain, while reactive oxygen species activate biosignaling pathways that cause cellular harm. Mechanical ventilation should therefore be administered more conservatively, especially in the case of treating COVID-19 patients. Several studies echo this warning, suggesting that tightly controlled, lower physiological PaO_2_ levels can mitigate both cardiac injury and lower mortality rates [173].

### 6.5. Recommendations for Physicians

While murine models provide the mechanistic blueprint for how hyperoxia affects cardiac and lung physiology, data from clinical settings such as COVID-19 and critical care cohorts underscore the risk of mortality and long-term remodeling. As summarized in Table 1, the supplemental oxygen or hyperoxia induced pathophysiology can impact long-term health outcomes, it is therefore important for the physicians to consider the administration of supplemental oxygen as a pharmacological intervention with a limited interval of effectiveness. Increased monitoring, for one, is a necessity to assess abnormalities associated with supplemental oxygen such as QT and QTc interval prolongation [13,14,15,17,20,21]. Clinicians should also aim to decrease the target oxygen concentration to more conservative standards to decrease the instances where ROS trigger their downstream metabolic pathways. In clinical settings, oxygen should be titrated to its lowest effective dose, and frequent reassesment and weaning are needed. By limiting exposure duration and concentration, clinicians mitigate oxidative strees induced lung edema and cardiac remodelling.

## 7. Limitations of Current Data

Review of contemporary literature has revealed certain weaknesses in current data and the conclusions that can be made regarding hyperoxia and its effects on cardiac and pulmonary physiology. For instance, a significant portion of the mechanistic data regarding hyperoxia-induced structural and electrical remodeling (such as ion channel downregulation and activation of inflammatory cytokines) originates from animal research. Extrapolating exact time-dose thresholds for hyperoxic injury from murine models to critically ill humans remain challenging, as human clinical outcomes are often confounded by primary disease processes. Furthermore, critically ill patients receiving mechanical ventilation and supplemental oxygen are rarely treated with these interventions in isolation. The relative contribution of hyperoxia versus concurrent pharmacological interventions to arrhythmogenesis in the ICU is difficult to isolate, particularly when patients are administered medications known to alter cardiac repolarization. Clinical reliance on load-dependent functional parameters such as ejection fraction may also lead to the under-recognition of hyperoxia-induced cardiac dysfunction. There is a need for broader clinical adoption of load-independent indices, such as LVOT VTI and TAPSE, to accurately assess hemodynamic status under positive-pressure ventilation. Another concern is that experimental data suggests hyperoxia leaves a persistent structural imprint on the myocardium, but longitudinal clinical studies are required to determine the long-term impact of temporary hyperoxic exposure on survivors’ subsequent risk for chronic heart failure.

## 8. Conclusions

In summary, supplemental oxygen remains an indispensable therapeutic intervention in modern medicine; however, its administration is not without significant cardiovascular consequences when delivered in excess. This review highlights that hyperoxia is a critical driver of cardiopulmonary dysfunction through interconnected mechanisms involving oxidative stress, inflammation, and mechanical perturbations introduced by mechanical ventilation. Elevated reactive oxygen species disrupt mitochondrial function, impair calcium handling, and activate pro-fibrotic and hypertrophic signaling pathways, collectively promoting structural remodeling of the myocardium. Simultaneously, alterations in intrathoracic pressure and pulmonary vascular resistance impose hemodynamic strain, particularly on the right ventricle, ultimately compromising cardiac output and systemic perfusion. These structural and functional changes are further compounded by electrophysiological remodeling, creating a substrate for arrhythmogenesis (Table 1). Importantly, emerging clinical evidence underscores that these effects are dose- and duration-dependent, reinforcing the need for precision in oxygen delivery. A paradigm shift toward conservative, titrated oxygen therapy is therefore essential to balance adequate tissue oxygenation with the minimization of hyperoxia injury. Future research should focus on defining optimal oxygenation thresholds, elucidating patient-specific susceptibilities, and developing targeted interventions to mitigate oxidative and inflammatory damage, thereby improving cardiovascular outcomes in critically ill populations.

## Figures and Tables

**Table 1:** **Comparative Pathophysiology of Respiratory Support and Oxygen Concentrations.**

Category	Driver	Lung Status	ROS/Stress	Cardiac Effect	Long-Term Risk
Normoxia (baseline)	21% O_2_/ATM pressure	Stable Alveoli (N_2_ scaffold intact)	Balanced/antioxidants sufficient (glutathione, superoxide dismutase)	Normal hemodynamics	Physiological baseline
Supplemental 02 (controlled)	FiO_2_ 21–60%	Blunted HPV reflex → V/Q mismatch ↑ Lung lavage protein (early injury) ↓ N_2_ → early alveolar instability ≥7 days at 50–60%: lung “primed” for toxicity ↑ Lung weights in inflammatory models (pulmonary edema)	Antioxidants sufficient at low FiO_2_, increasingly stressed approaching 60%	Vagus-mediated ↓ HR → ↓ cardiac index, ↓ Stroke volume, ↑ Pulmonary capillary wedge pressure, Hemodynamic strain in heart failure patients	O_2_ toxicity, V/Q mismatch, hemodynamic compromise
Mechanical Ventilation (Pressure)	Positive pressure/PEEP	N_2_ washout → absorption atelectasis, Alveolar overdistension → capillary compression, VILI → biotrauma (TNF-α, IL-6 released systemically)	Mechanical strain, Neurohormonal activation: sympathetic ↑, RAAS (angiotensin II, aldosterone), Catecholamine-driven ↑ Ca^2+^ cycling	↓ Venous return/preload, ↑ RV afterload (↑ PVR), Septal shift → ↓ LV filling, Diastolic dysfunction (stiff ventricle → output-limited), EF preserved but stroke volume low—EF misleading in MV, Angiotensin II → hypertrophic gene expression, Aldosterone → collagen synthesis	RV dilation, Interstitial fibrosis (ECM expansion, reversal limited once established), Electrical instability, Progressive structural remodeling
Hyperoxia (Pathological)	FiO_2_ > 90%	Lipid peroxidation → Type I & II alveolar cell destruction, Surfactant dysfunction → ↑ surface tension, ↓ compliance, HALI → ARDS-like injury → pulmonary fibrosis, Mortality in animal models at 72–96 h	Massive ROS, ↓ Nitric oxide → endothelial dysfunction, NF-κB → myofibroblast differentiation, Mitochondrial dysfunction → ↓ ATP, SERCA↓ + RyR leak → Ca^2+^ dysregulation	↓ Cardiac output & ejection fraction, Bradycardia, ↑ QTc & JT intervals, Nav1.5↓ → slowed conduction → reentry, K^+^ channel remodeling → repolarization dispersion, Ca^2+^ dysregulation → delayed afterdepolarizations, Septal shift/RV failure, ROS → myofibroblast activation	Interstitial fibrosis, Arrhythmogenesis, Reversal increasingly limited once fibrosis established, AKI/multi-organ dysfunction (lung–heart–kidney axis), COVID-19: 31% de novo RV dysfunction post-intubation, MV = largest independent mortality risk factor (beyond age > 80)

**Symbols:** ↑, increase or elevation; ↓, decrease or reduction; →, leading to or resulting in.

## Data Availability

No new data was created or analyzed in this study. Data Sharing is not applicable to this article.

## Abbreviations

The following abbreviations are used in this manuscript:

AKI	Acute kidney injury
ARDS	Acute respiratory distress syndrome
AT1	Angiotensin II type 1 receptor
ATP	Adenosine triphosphate
COPD	Chronic obstructive pulmonary disease
COVID-19	Coronavirus disease 2019
ECM	Extracellular matrix
EF	Ejection fraction
FiO_2_	Fraction of inspired oxygen
HALI	Hyperoxic acute lung injury
HDLVEF	Hyperdynamic left ventricular ejection fraction
HFNC	High-flow nasal cannula
HPV	Hypoxic pulmonary vasoconstriction
ICU	Intensive Care Unit
IL-6	Interleukin-6
I_K1	Inward rectifier potassium current
I_Kr	Rapid delayed rectifier potassium current
I_Ks	Slow delayed rectifier potassium current
I_Na	Fast inward sodium current
I_to	Transient outward potassium current
LV	Left ventricle/left ventricular
LVOT VTI	Left ventricular outflow tract velocity–time integral
MAP	Mean arterial pressure
MI	Myocardial infarction
mRNA	Messenger RNA
MV	Mechanical ventilation
Nav1.5	Voltage-gated sodium channel isoform 1.5
NF-κB	Nuclear factor kappa B
NFAT	Nuclear factor of activated T-cells
NIV	Non-invasive ventilation
PaO_2_	Partial pressure of arterial oxygen
PEEP	Positive end-expiratory pressure
PI3K–Akt	Phosphoinositide 3-kinase-protein kinase B pathway
PVR	Pulmonary vascular resistance
RAAS	Renin–angiotensin–aldosterone system
ROS	Reactive oxygen species
RV	Right ventricle/right ventricular
RyR	Ryanodine receptor
SaO_2_	Arterial oxygen saturation
SCN5A	Gene encoding Nav1.5 sodium channel
SERCA	Sarco/endoplasmic reticulum calcium ATPase
SpO_2_	Peripheral oxygen saturation
T1DM	Type 1 diabetes mellitus
TAPSE	Tricuspid annular plane systolic excursion
TNF-α	Tumor necrosis factor alpha
VILI	Ventilator-induced lung injury
V/Q	Ventilation–perfusion
